# Temporal regulation of genetic programs governing multiple cell death during myocardial ischemia-reperfusion injury

**DOI:** 10.3389/fgene.2025.1632867

**Published:** 2025-09-05

**Authors:** Qiuyu Pang, Xiangmin Meng, Zhenfang Zhou, Lu You, Jinghan Yuan, Qipu Feng, Bingmei Zhu

**Affiliations:** ^1^ Regenerative Medicine Research Center, West China Hospital, Sichuan University, Chengdu, China; ^2^ Animal Experiment Center, West China Hospital, Sichuan University, Chengdu, Sichuan, China

**Keywords:** myocardial infarction, reperfusion, programmed cell death, ferroptosis, apoptosis

## Abstract

**Introduction:**

Reperfusion serves as an effective therapeutic strategy for myocardial infarction (MI), but it causes damage to the heart. Although many studies have investigated the mechanism of disparate forms of cell death in myocardial ischemia-reperfusion injury (I/R), there remains a paucity of studies focus on the direct comparison of the mode of cell death events resulting from different reperfusion periods.

**Methods:**

We conducted an analysis of different sequencing data available in public databases to investigate the relationship between the diverse patterns of cell death and different reperfusion times. Additionally, we evaluated the time window of multiple categories of cell death between cells and mice.

**Results:**

We explored the relationship between the various modes of cell death and different reperfusion times induced by 6h, 12h and 24 h reperfusion. Our findings revealed that apoptosis occurred in the early stage of I/R injury and continued to appear as the reperfusion time increased. Meanwhile, the changes in autophagy and cuproptosis were also more obvious in the early stage of reperfusion. Notably, ferroptosis and necrosis emerged as the predominant forms of cell death during the medium-to-long-term reperfusion period.

**Discussion:**

In summary, this study demonstrates that apoptosis takes place during the early stage of reperfusion. Besides, ferroptosis, necrosis and pyroptosis played a crucial role in the prolonged I/R injury period.

## 1 Introduction

Coronary artery disease (CAD) remains a leading cause of morbidity and mortality worldwide (Hausenloy and Yellon, 2013; Heusch, 2013). When myocardial blood supply is interrupted, percutaneous coronary intervention (PCI) and thrombolytic therapy are the most widely utilized therapeutic strategies for achieving timely and effective myocardial reperfusion in eligible patients (Ibáñez et al., 2015). Timely reperfusion resupply of blood to affected tissue can reduce myocardial infarct size, nevertheless, reperfusion is also capable of triggering irreversible damage to the myocardium, known as myocardial ischemia-reperfusion (I/R) injury (Heusch, 2020). The occurrence of I/R injury is due to a complex set of reasons. Multiple mechanisms contribute to cardiac I/R injury, including oxidative stress (Piper et al., 2003), excessive reactive oxygen species (ROS) generation (Cadenas, 2018), Ca^2+^ overload (Zhou et al., 2017), inflammation and mitochondria dysfunction (Inserte et al., 2012). Reperfusion can cause multiple categories of cardiomyocyte death, like apoptosis (Del Re et al., 2019), ferroptosis, autophagy, necrosis (Cai et al., 2023), and pyroptosis (Shi et al., 2021), eventually leading to myocardial remodeling, along with reduced myocardial contractility and cardiac function (Mishra et al., 2019). An intricate interplay exists among different forms of cell death. These multiple forms of cell death can occur concurrently, interact with each other, and collectively contribute to the complexity of myocardial I/R injury. It is plausible that inhibitors targeting different cell death modalities may exhibit a synergistic effect in the treatment of myocardial I/R injury. However, despite the progress of the treatment approaches against the I/R injury has yet been achieved according to these mechanisms, the time point where diverse patterns of cell death takes place following reperfusion injury is still unclear. Notably, targeted manipulation of molecules involved in specific cell death processes at the appropriate reperfusion time point could exert a notable effect on mitigating I/R injury.

Given the limited regenerative capacity of cardiomyocytes, myocardial I/R injury is strongly associated with programmed cell death (PCD) of terminally differentiated cardiomyocytes, which act as key contributors to the pathophysiological process of myocardial I/R injury (Whelan et al., 2010). In accordance with inducing stresses and regulatory signaling pathways, internal apoptosis is regarded as an outstanding regulatory procedure, caused by destroyed mitochondria as well as mediated by caspase3, caspase9, BAX, BCL2, whereas excessive apoptosis can accelerate the pathological progression of myocardial I/R (Tang et al., 2019). Indeed, cell necrosis is invariably seen as an uncontrollable pattern of PCD, nonetheless, according to recent research, several categories of cell necrosis, like pyroptosis, ferroptosis and necroptosis, are also amenable to modulation (Galluzzi et al., 2018). It is mainly triggered by calcium overload and ROS overproduction, and PARP1 and AIF are important effector molecules. Ferroptosis is a novel category of iron-dependent programmed PCD, which is different from autophagy and apoptosis and directly triggered by lipoxygenase (LOX) (Jiang et al., 2021). Glutathione peroxidase 4 (GPX4), glutathione peroxidase 4 (ACSL4), solute carrier family seven member 11 (SLC7A11), and transferrin receptor1 (TFR1) were key regulators. ACSL4 and TFR1, as promoting-ferroptosis molecules, promote the generation of lipid peroxidation and iron ion transport, which can decompose to form additional ROS, further exacerbating oxidative stress (DeGregorio-Rocasolano et al., 2019). GPX4 and SLC7A11 are key inhibitor of ferroptosis, GPX4 acts to scavenge membrane lipid hydroperoxides. However, when GPX4 is inactivated or when GSH is depleted, peroxides accumulate within the cell, triggering ferroptosis (Du et al., 2025). SLC7A11 facilitates the import of cystine from the extracellular environment into the cell and synthesizing GSH. Inhibition of SLC7A11 activity or expression can lead to a depletion of GSH and an accumulation of ROS, ultimately inducing ferroptosis (Badgley et al., 2020). Cells undergoing ferroptosis present a special mitochondrial morphology. Xu Zhang et al. found that Alox15/15-HpETE (15-lipoxygenase/15-Hydroperoxyeicosatetraenoic acid)–mediated cardiomyocyte ferroptosis plays a crucial role in prolonged I/R injury (Cai et al., 2023). Pyroptosis is a form of programmed cell death mechanistically dependent on the activation of the NLRP3 inflammasome complex, which subsequently induces the proteolytic activation of caspase-1 and the specific cleavage of gasdermin D (GSDMD) at its interdomain linker region. Morphologically, pyroptosis is characterized by plasma membrane pore formation and the secretion of proinflammatory cytokines such as IL-1β and IL-18. Both GSDME and GSDMD are key proteins involved in the pathogenesis of pyroptosis. (Chen et al., 2016). Autophagy, a highly conserved cellular degradation process, is precisely regulated by the mechanistic target of rapamycin (mTOR) and AMP-activated protein kinase (AMPK) signaling pathways, which serve as central regulators of cellular energy homeostasis. The autophagic machinery involves two essential protein complexes: Beclin-1, a critical component of the class III phosphatidylinositol 3-kinase (PI3K) complex, and microtubule associated LC3, which plays a pivotal role in autophagosome membrane formation and elongation. Notably, autophagy exhibits a dual role in cellular fate determination while basal or moderate autophagy acts as a cytoprotective mechanism by maintaining cellular homeostasis and preventing cell death, excessive or dysregulated autophagy can induce autophagic cell death and tissue damage through the over-degradation of essential cellular components. Recent studies have revealed that cardiomyocyte pyroptosis contributes to myocardial ischemia-reperfusion (I/R) injury (Shi et al., 2021), however, few studies focus on the direct comparison of the mode of cell death events resulting from different reperfusion periods. A comprehensive understanding of the dominant phases of various myocardial cell death modalities following myocardial I/R injury would be critical for developing and selecting appropriate therapeutic strategies at different reperfusion time windows.

In this study, we investigated the relationship between the various modes of cell death and different reperfusion times based on available bulk sequencing data. The marked progresses of single-cell sequencing technique make extensive utilization of single-cell analysis correctly depict each cell structure in myocardial I/R injury and deeply construe the activity of signaling pathways in terms of a cell type. We then analyzed the cell death of which cell type appeared using Single cell RNA-Seq (scRNA-Seq) datasets. Additionally, through the analysis of the Spatial-RNA sequencing (ST-seq) data, we mapped the spatial distribution of different cell death modalities within the heart. Furthermore, we validated these analytical findings using both cellular and animal models. Our study might offer a well-defined relationship between the various modes of cell death and different reperfusion periods and may provide a potential new target for clinical treatment. By enhancing understanding of the mechanisms and temporal regulation among different types of cell death in myocardial I/R injury, this study aims to pave the way for the development of novel interventions for cardio-protection in patients affected by myocardial I/R injury.

## 2 Materials and methods

### 2.1 Animals

All Male C57BL/6 mice aged 8–10 weeks were purchased from GemPharmatech Co. Ltd., (Chengdu, China) and raised at the specific pathogen-free laboratory animal facility of West China Hospital, Sichuan University (Chengdu, China). All efforts were made to minimize suffering in animals. All mice were maintained under a 12-h light/dark cycle at 22 °C before initiation of the experiments. Mice were randomly assigned to groups, with cohousing of mice within the same group at a density of ≤5 per cage. At the end of the experiment, mice were anaesthetized with 2% isoflurane and euthanized by cervical dislocation. The total protocols concerning animals were implemented according to the associated moral guide-lines. The experiment design received approvals from the Animal Care and Use Committee of the West China Hospital of Sichuan University (Chengdu, China) (No. 20240524005).

### 2.2 Animal model of I/R

8-week male mice were applied to build the I/R animal model. Animals were immobilized to 1 mouse plate at 37 °C. Moreover, the precordial area was disinfected with iodophor. All the mice were subjected to inhalation anesthesia using 2% isoflurane before the surgery. Left anterior descending coronary artery (LAD) was tied to a surgical slipknot by a 7–0 silk suture to create the ischemia mice model. Sham-operated mice underwent the same anesthetic administration and thoracotomy exposure without occlusion of the LAD. The sham-operated control group were sacrificed after 6 h reperfusion. Electrocardiography was implemented to assess the excellent production of the ischemia model. After half an hour, the slipknot for cardiac reperfusion was unfolded. Mice were euthanized at designated time points, and myocardial tissues were collected for comprehensive analysis.

### 2.3 Cell culture and hypoxia/reoxygenation (H/R) treatment

H9c2 cells were obtained from Procell Life Science Technology Co., Ltd. (Wuhan, China) and cultured in 4.5 g/L high-glucose DMEM medium in a CO_2_ incubator (37 °C, 5% CO_2_). When cell confluency reached approximately 90%, cells were passaged or plated for subsequent experiments. For H/R treatment, the H9c2 cells were cultured for 8 h in glucose-deprived DMEM under hypoxic conditions using a three-gas low oxygen constant temperature incubator (Esco Lifesciences Group, Singapore) with 5% CO_2_, and 1.1% O_2_. Reoxygenation was performed by culturing the H9c2 cells in DMEM containing 10% FBS for 2h, 6h, 12h, 24 h in an incubator with 5% CO_2_and 95% air respectively.

### 2.4 Electrocardiogram

Electrocardiogram (ECG) was executed instantly after the completion of ligation. All mice were anesthetized with 2% isoflurane and meticulously placed on the ECG platform to obtain surface lead II ECG. After the lead settings and system adjustments were completed, electrocardiograms were recorded. Then the results were analyzed with the LabChart 8.2.3 software (AD Instruments, Australia).

### 2.5 Western blotting

For total protein extraction, the heart ischemic area tissues were dissolved within the RIPA buffer comprised of a complete protease and phosphatase suppressor cocktail (New Cell and Molecular Biotech) (Sung et al., 2024). Protein density was evaluated through the bicinchoninic acid (BCA) assay kit (23,225, Thermo Fisher Scientific). After that, the proteins were segregated via precast SDS–PAGE gel (4%–20%, YEASEN). In addition, bands were electro-transferred onto PVDF membranes (03010040001, Merck). The PVDF membranes were blocked with protein free rapid blocking buffer (20 min) and incubated with the primary antibody overnight at 4 °C (Supplementary Table S1 offers the antibody information). After rinsing three times through the TBST, the PVDF membranes were incubated with the secondary antibody for 1 h at room temperature. All protein bands were monitored using ECL buffer (32,209, Thermo Fisher Scientific). Western blotting quantifications were performed using ImageJ software (NIH, Bethesda, MD, United States; http://rsb.info.nih.gov/nih-image/).

### 2.6 RT-qPCR

The total RNAs were obtained from heart ischemic area tissues or cells through the Cell/Tissue Total RNA Kit (19221ES50, YEASEN). After cDNA synthesis by means of Hifair^®^ one-step RT-gDNA digestion SuperMix for qPCR Kit (11142ES60, YEASEN). Real-time PCR amplification was implemented through the ChamQ Universal SYBR qPCR Master Mix (11201ES08, YEASEN) on ABI QuantStudio6 Q6 Real-time PCR system (ABI, United States). The comparative expression of mRNA was computed through ΔΔCt approach in light of standard methods. Supplementary Table S2 display the gene-specific primer sequences.

### 2.7 Immunofluorescent staining for cardiac tissues

The mice were euthanized, and the heart tissue was washed with PBS. For immunofluorescence staining, ischemic area tissue was placed on an optimal cutting temperature (OCT) compound and immediately froze, then 10 μm cryo sections were generated (Choudhury et al., 2011; Alayoubi et al., 2024). Cryosections were immobilized through 4% paraformaldehyde, permeabilized with 0.5% Triton X-100, and blocked with 2% bovine serum albumin, and then incubated with the primary antibody GPX4 (ET1706-45, HUABIO, 1:100 dilution) overnight at 4 °C. The sections were washed thrice with PBS for 5 min each and incubated with the secondary antibodies for 1 h at room temperature. Fluoroshield histology mounting medium with DAPI was used to cover the slides, and all sections were explored through the confocal microscope (ECLIPSE Ti, Nikon).

### 2.8 TUNEL staining

Myocardial ischemic area tissue apoptosis was assessed using the TUNEL BrightRed Apoptosis Detection Kit (A113-01, Vazyme) following the manufacturer’s instructions. Briefly, the 5 μm frozen heart sections were washed thrice with PBS for 5 min each and fixed with 4% formalin for 15 min at room temperature. The TUNEL working solution was added and the slides were placed in an incubator at 37 °C for 1 h. The slides were washed thrice with PBS for 5 min each, and coverslips were mounted with DAPI containing mounting medium. All sections were examined and photographed using a confocal fluorescence microscope.

### 2.9 CCK-8 assay

Cardiomyocyte viability was assessed using CCK-8 Assay Kit (40203ES60, YEASEN) as per the manufacturer’s guidance. H9c2 cells were seeded into 96-well plates and cultured. Subsequently, the medium in each well was replaced with DMEM containing 10% CCK8 reaction solution. To be specific, cells were nurtured for 1 h. Finally, cell activity was evaluated at 450 nm through one microplate reader.

### 2.10 Analysis of single-cell RNA-sequencing data

#### 2.10.1 Data resource and RNA-seq data analysis

RNA-sequencing data GSE193997 (Liu et al., 2022), CRA008354 (Ni et al., 2023), GSE168610 (Eijgenraam et al., 2021) were downloaded from the Gene Expression Omnibus (GEO) (https://www.ncbi.nlm.nih.gov/geo) and Genome Sequence Archive (GSA) (https://ngdc.cncb.ac.cn/gsa/). The comprehensive description of the publicly available datasets was summarized in Supplementary Table S3. In order to obtain the high-quality gene sets of different programmed cell death (PCD), we collected the genes from the published literature and databases, including MSIGDB, AMIGO2, Ferrdb and KEGG. At last, our study incorporated autophagy (n = 663), apoptosis (n = 250), ferroptosis (n = 474), cuproptosis (n = 14), necroptosis (n = 159), necrosis (n = 549), pyroptosis (n = 201) (Xie et al., 2023; Tsvetkov et al., 2022; Castanza et al., 2023; Kanehisa and Goto, 2000; Zhou et al., 2023). To mitigate the influence of different RNA-seq analysis pipelines, we downloaded raw data from GEO. At first, the adaptors from the raw data were removed by “Trimgalore” (v.0.6.7). Trimmed reads were mapped to the genome (mm10) with Hisat2 (v 2.2.1) (default parameters). Mapped reads were quantified to the exon level (-t exon) through the “featureCounts” (v 2.0.6). We applied Transcripts Per Kilobase Million (TPM) to normalize reads. Principal Component Analysis (PCA) was analyzed according to “FactoMineR” and “factoextra” R packages. We analyzed the PCD score at different time points using the “GSVA” R package (version 1.46) with the following parameters: (kcdf = “Gaussian” and method = “ssgsea”). We visualized the results using the “ggplot2” R package (version 3.4.2) after z-score normalization.

### 2.11 scRNA-seq data analysis

ScRNA-Seq data GSE227238 were analyzed using the “Seurat” R package (version: 4.3, https://satijalab.org/seurat/). Based on the data characteristics, quality control was performed as follows: cells with fewer than 200 detected features or a mitochondrial UMI percentage exceeding 10% were filtered out. The function “NormalizeData” and “scaleData” were used for normalization and scaling. At first, we filtered doublet cells using R package “DoubletFinder” (version 2.0.3). Additionally, we removed the data for cells with contamination scores greater than 20% using R package “DeconX” (version 0.99). All samples were integrated using function “IntegrateData”. PCA was analyzed in light of the scaled data through the top 2,000 escalated variable genes. Then, the top 30 principal components with the highest variance were utilized for dimensionality reduction and clustering using “RunPCA”, “UMAP”, and “TSNE” functions. For annotation of clustering, we combined reference data from CellMarker2 and published literature for cell annotation (Lin et al., 2023). To calculate the PCD score, the AddModuleScore function was used. Finally, data visualization was performed using the R packages “ggplot2” and “scRNAtoolVis”.

### 2.12 Spatial-RNA sequencing (ST-seq) data analysis

ST-seq data GSE227088 (Lin et al., 2023) were analyzed using “Load10X_Spatial” function from “Seurat” R packages. The Seurat’s “AddModuleScore” function was used to calculate PCD score of ST-seq data.

### 2.13 Statistical analysis

Data from mice and cell models were denoted as mean ± SD. Remarkable differences were evaluated through Independent-sample t-test (two-tailed) for statistical contrasts between 2 groups, or through One-way ANOVA before Tukey *post hoc* test for statistical contrasts among multiple groups. The value of P < 0.05 reached statistical difference. Analyses were implemented through GraphPad Prism 8.

## 3 Results

### 3.1 Identification of gene expression patterns between 7 forms of cell death modes and reperfusion time points

**FIGURE 1:**
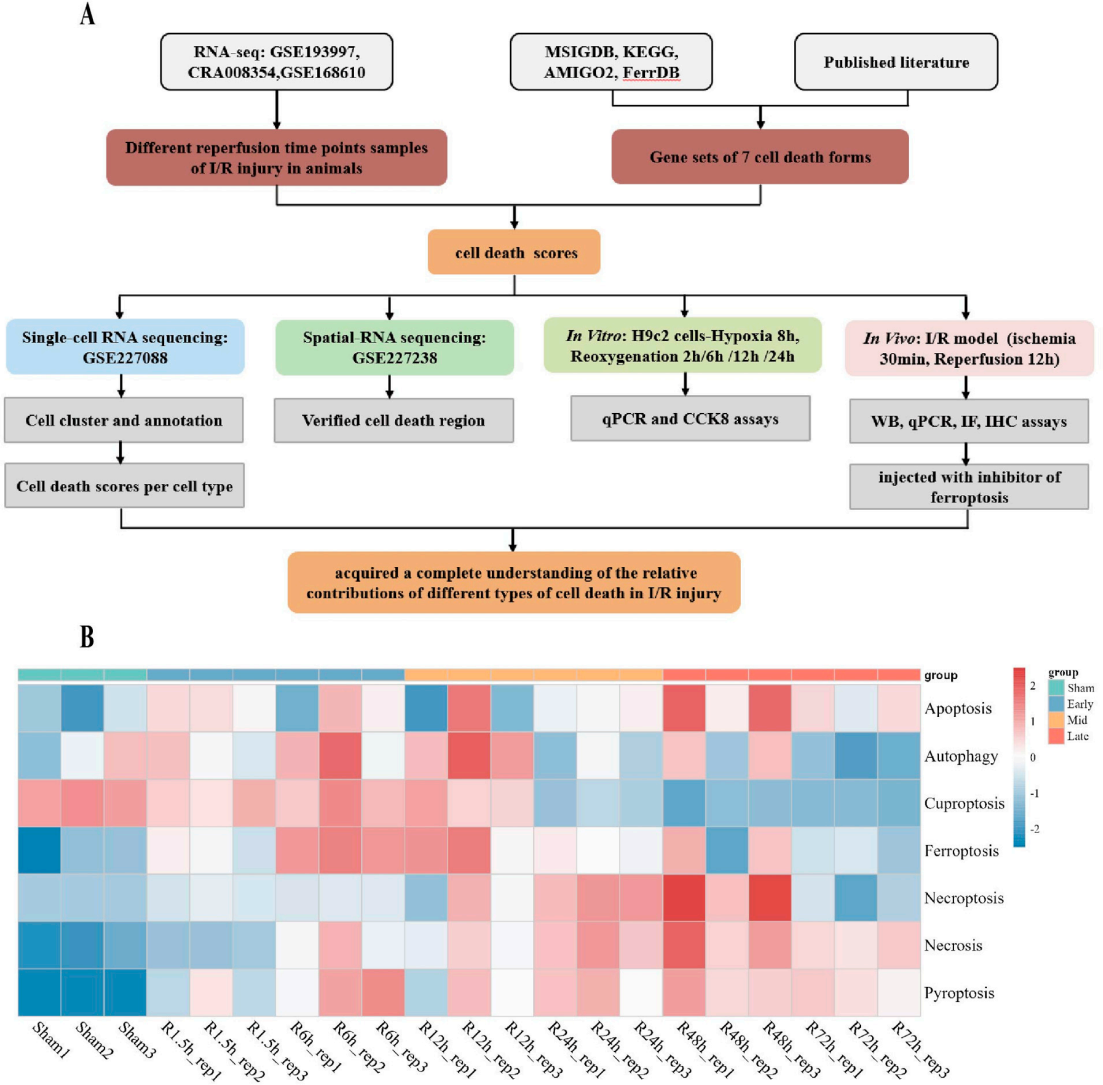
Establishment of the relationship between seven discrepant cell death forms and reperfusion times. **(A)** The research flowchart. **(B)** Genes regarding diverse cell death forms were analyzed including apoptosis, autophagy, cuproptosis, ferroptosis, necroptosis, necrosis, pyroptosis and analyzed the expression and results of multivariable reperfusion time points. (GSE193997 GSE193997 included three time points: 1.5 h of reperfusion (R1.5 h), 6 h of reperfusion (R6h), and 12 h of reperfusion (R12 h)., CRA008354 included two time points and sham groups: 24 h of reperfusion (R24 h), 48 h of reperfusion (R48 h), the reperfusion time of GSE168610 is 72 h (R72 h), each reperfusion time point and sham groups has three biological replicates, n = 3.).

To comprehensively clarify the comparative contributions of discrepant categories of cell death in myocardial I/R injury, we downloaded multiple RNA-sequencing data obtained at different reperfusion time points (GSE193997, CRA008354, GSE168610) from the GEO and GSA. The study flowcharts are shown in Figure 1A. In terms of seven cell death forms, like pyroptosis, apoptosis, autophagy, cuproptosis, ferroptosis, necroptosis and necrosis, their signature gene sets were gathered from released databases and literature. On the time axis, we attempt to evaluate cell death form changes in cell architecture following ischemic injury (I-0h), along with in the early (R-1.5h, R-6h), middle (R-12h, R-24 h), and late (R-48h, R-72 h) phases of reperfusion (Figure 1B).

Based on the gene expression of selected gene sets for 7 cell death forms, we identified that each cell death form exhibited distinct characteristics that reflected the progression of I/R injury.

Cardiomyocyte death is a direct consequence of I/R injury, with significant loss of cardiomyocytes initiating at R-6h. For the early stage, substantial myocardial apoptosis and ferroptosis were significantly increased after 6 h post reperfusion. Followed by the middle stage, pyroptosis and ferroptosis were the dominant shape of PCD within the medium duration frame following I/R. As expected, necroptosis and necrosis began at R-12 h and achieved important levels through R-48h, and of note, necrosis persisted for a longer period of time. Furthermore, our data revealed that autophagy and cuproptosis appeared in the early stage but reduced in the medium-to-long-term time, ferroptosis and necrosis may be involved in the prolonged stages of I/R injury (Figure 1B).

### 3.2 Single-cell phenotyping of cell death forms in myocardial I/R injury

**FIGURE 2:**
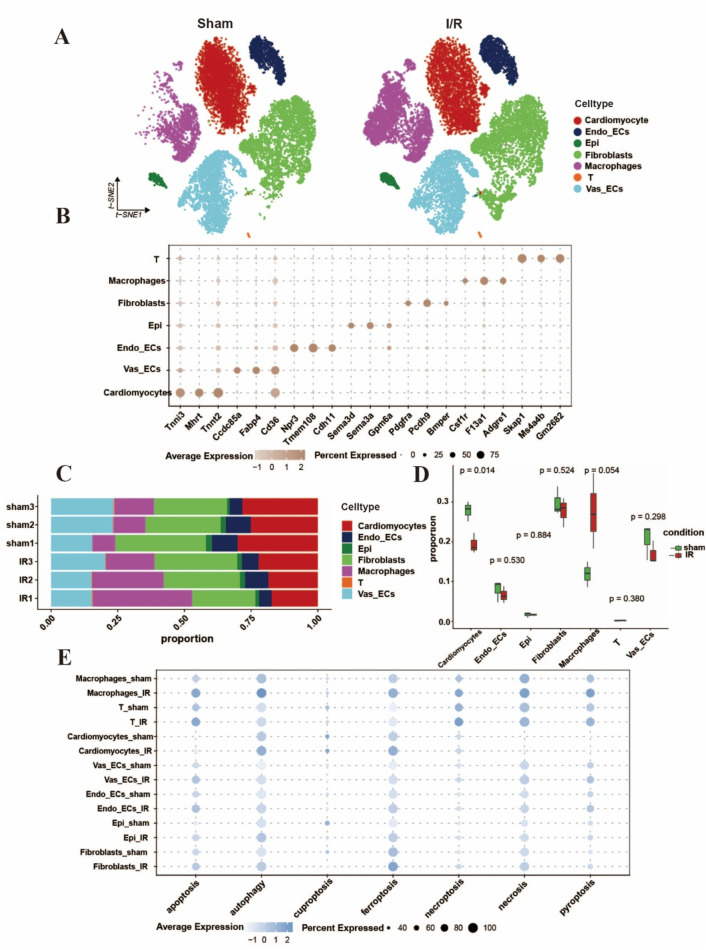
Single-cell characterization of cell death forms in Myocardial I/R injury. **(A)** UMAP diagram of cell type distribution according to the pooled scRNA-Seq data per group tissues. **(B)** The bubble plot showing the marker genes regarding cell clusters and categories, the bubble size stands for the ratio of cells showing marker genes. **(C,D)** Histograms displayed the proportions of discrepant cells throughout the cardiac tissues in each group. **(E)** The bubble plots indicated the seven different cell death forms of major cell categories, separately. The dot size was in proportion to the measured contributing fraction in cell death forms. (Established the myocardial I/R injury model by occluding the LAD for 30 min of mouse, reperfusion process for 24 h).

Cardiac tissue comprises a diverse array of cell types, and the aforementioned analyses were based on bulk transcriptomic data. These findings provided comprehensive information about various cell types, yet they failed to depict cell diversity at the single-cell resolution level. To better simultaneously depict the single cell PCD and elucidate signaling patterns before and after I/R injury, we downloaded scRNA-Seq data GSE227238 and further analyzed the influence of I/R on cell types and transcriptional modes among cardiac tissues. Following quality control, batch effect correction, normalization, and clustering of the sequencing data, we identified key canonical marker genes and annotated the major cell types (Litvinukova et al., 2020; Kalucka et al., 2020), including endothelial cells, fibroblasts, cardiomyocytes, T cells, and macrophages. These results were consistent with previously published findings on molecular profiles and cellular composition (Lin et al., 2023; Farbehi et al., 2019) (Figures 2A,B). In contrast to the sham group, the I/R group exhibited an evident decrease in cardiomyocytes, while the ratio and count of macrophages and fibroblasts rose following myocardial I/R injury (Figures 2C,D). We next extrapolated the cell death of which cell type appeared. Gene expression analysis revealed distinct upregulation patterns of the 7 cell death modalities across different cell types (Figure 2E). For instance, after the continued reperfusion process for 24h, the cardiomyocytes showed obvious ferroptosis which was also consistent with the study by Cai et al. (2023).

### 3.3 Identification the influence of hypoxia/reoxygenation (H/R) on PCD of cardiomyocytes

**FIGURE 3:**
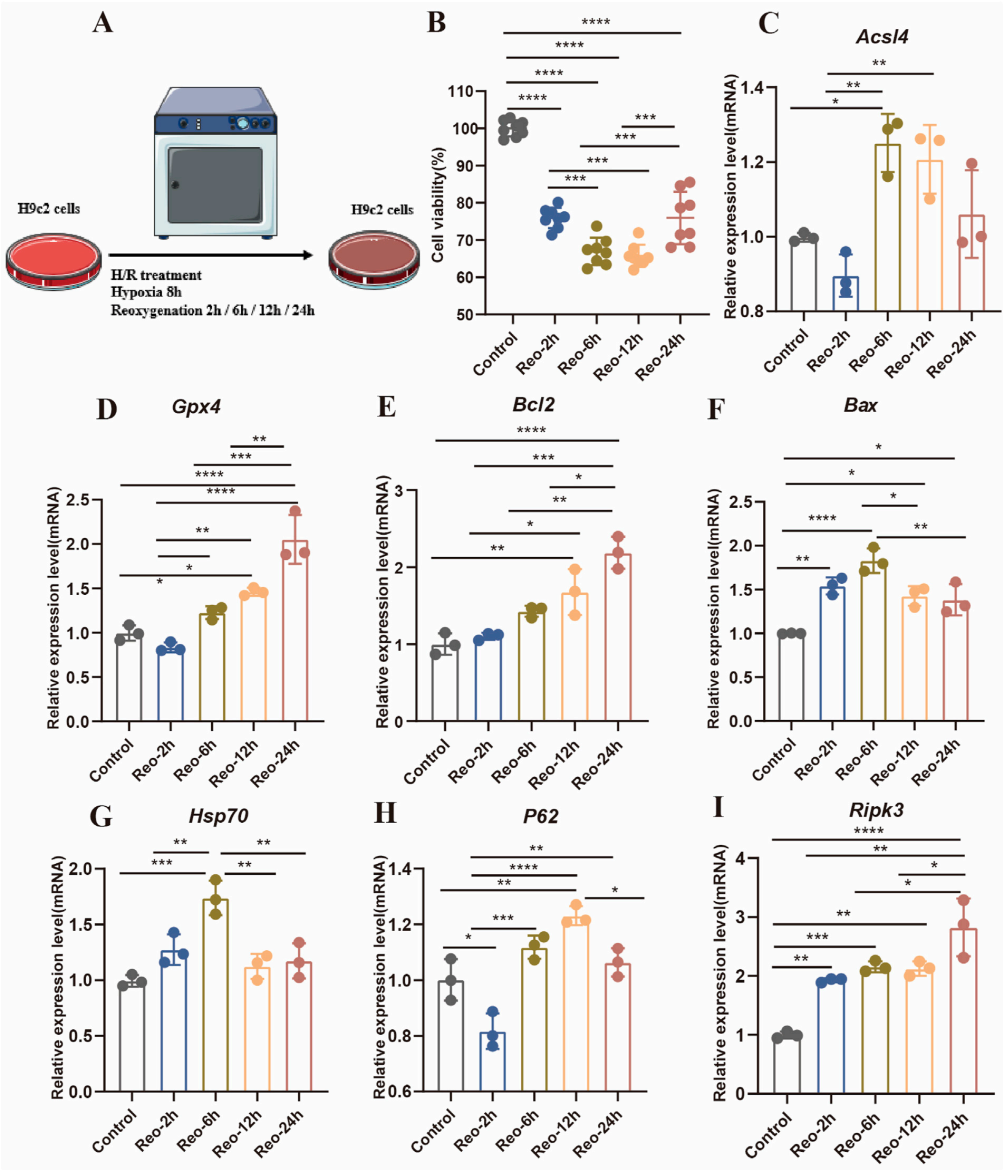
**(A)** Experimental protocol for *in vitro*. **(B)** Cell viability was detected by reoxygenation treatment with different times (n = 8: 8: 8: 8: 8). **(C–I)** Detection of gene expression levels associated with different modes of death (n = 3: 3: 3: 3: 3).

Continuous reperfusion therapy can exert impacts on various cell types within the heart. For instance, it can lead to endothelial cell dysfunction, cardiomyocyte necrosis, macrophage polarization, and fibroblast proliferation. Due to cardiomyocytes losing the endogenous regenerative capacity after birth, it was very important to determine the PCD time window of cardiomyocytes. we therefore investigated the H9c2 treated with H/R *in vitro* (Figure 3A). After H/R treatment, cell viability was significantly reduced, with a slight increase observed after 24 h of reoxygenation (Figure 3B). The abundance of apoptosis peaked at 6 h of reoxygenation (Figures 3E,F), autophagy is a dynamic process, and the related gene *P62* increased at 2 h after H/R treatment but decreased at 12 h (Figure 3H), With the delay of H/R treatment time*, Hsp70* reached its highest value at 6 h (Figure 3G), the expression of necrosis-related genes gradually increased (Figure 3I).

### 3.4 Time window of cardiomyocytes death during I/R injury in mice

**FIGURE 4:**
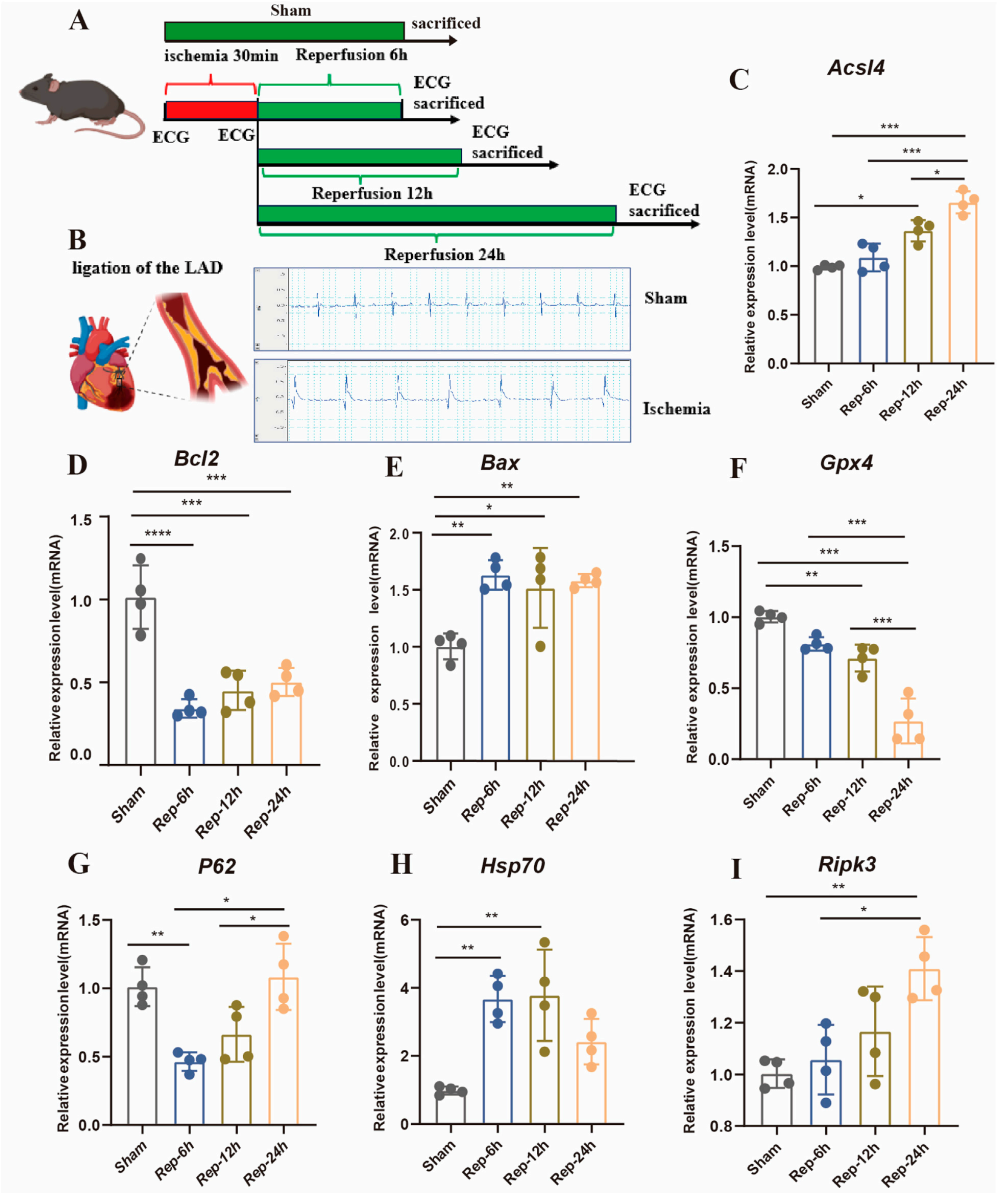
**(A)** Experimental design of myocardial I/R injury in mice. Myocardial I/R injury was caused by ligation of the LAD for half an hour, and then reperfusion for different time points (sham, R-0h, 6h, 12h and 24 h). **(B)** Typical ECG tracings per mouse group. **(C)** Representative images of HE, Scale bar:500 μm. **(D–I)** Detection of gene expression levels associated with different modes of death in mice (n = 4: 4: 4: 4).

**FIGURE 5:**
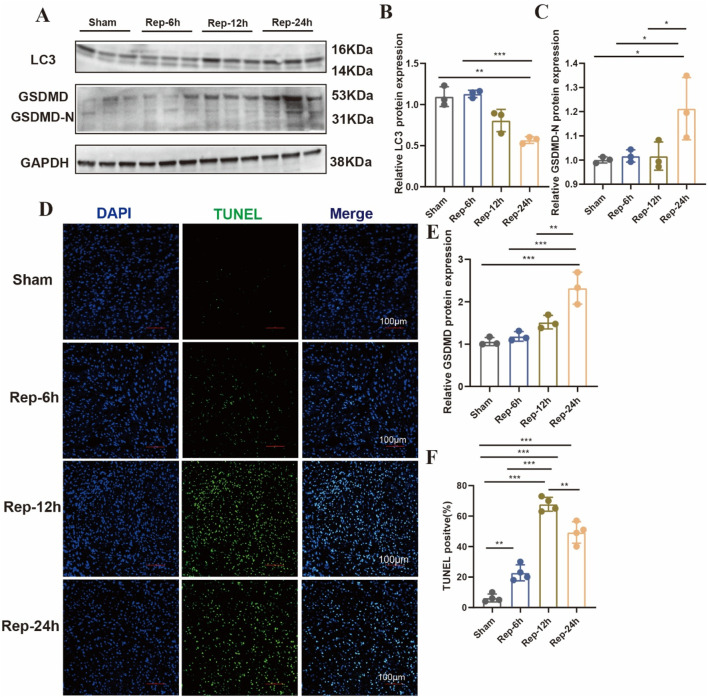
**(A)** Typical blot and **(B,C,E)** statistical data of myocardium different modes of death expressions among mouse hearts processed as illustrated (n = 3: 3: 3: 3). **(D)** Typical images of immunofluorescent staining and **(F)** quantification of the TUNEL active region among mouse hearts (n = 4: 4: 4: 4), Scale bar:100 μm.

Accumulating evidence indicates that multiple cell death modalities, including apoptosis, autophagy, cuproptosis, ferroptosis, necroptosis, necrosis, and pyroptosis, are involved in the progressive process of I/R injury (Del Re et al., 2019; Heusch, 2020). However, what role of various cell death played in discrepant phases of such a process is still unsure. Our study demonstrates that the gene expression levels were connected to disparate modes of death in myocardial tissue of mice. As shown in Figure 4, these results suggested that the PCD modes, like autophagy and apoptosis might participate in the early phases of I/R injury. In addition, ferroptosis, necrosis and pyroptosis is the dominant shape of PCD in the medium-to-long-ranging time process of I/R. Furthermore, TUNEL-positive signal was detected in myocardial tissues of the I/R group after 6 h of reperfusion (Figures 5D,F). The autophagy-related molecule LC3 showed the opposite trend (Figures 5A,B). It is worth noting that pyroptosis occurred gradually with the increase of reperfusion time (Figures 5C,E). These results are consistent with those in cells.

### 3.5 Ferroptosis is the predominant form of cell death in the medium-to-long-term time phase of I/R injury

**FIGURE 6:**
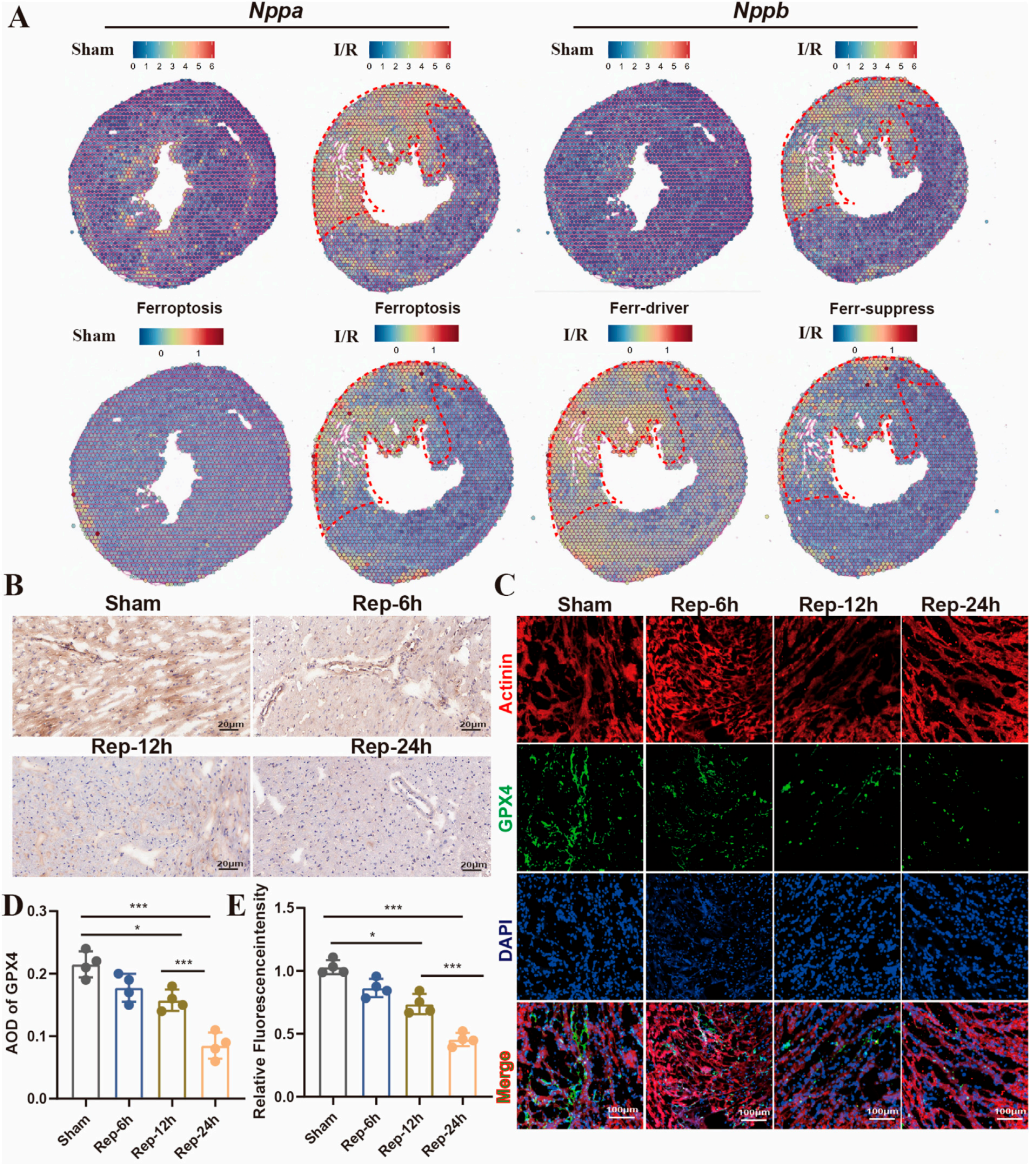
**(A)** Predicting the spatial position of ferroptosis-promoting, spatial transcriptome data impeded genes on heart slides (GSE227088, established the myocardial I/R injury model by occluding the LAD for 30 min of mouse, reperfusion process for 24 h). **(B,D)** Representative images and quantitative analysis of GPX4 immunohistochemical staining of heart sections at 6h, 12h, 24 h after reperfusion (n = 4: 4: 4: 4), Scale bar:100 μm. **(C,E)** Typical images of immunofluorescent staining and quantification of the GPX4 active cardiomyocytes among mouse hearts processed as illustrated (n = 4: 4: 4: 4) Scale bar:100 μm.

Ferroptosis is a crucial category of cardiomyocyte death from the infarction-reperfusion, particularly during the later stage of reperfusion (Cai et al., 2023). Furthermore, spatial transcriptome sequencing data were utilized to analyze the expression profiles of ferroptosis-promoting and ferroptosis-inhibiting genes in different regions of cardiac tissues. Firstly, we utilized *Nppa* and *Nppb* as molecular markers to precisely localize the cardiac injury regions (Figure 6A, top panels). Compared with the sham group, genes driving ferroptosis were significantly upregulated in the left ventricle ischemic areas after reperfusion 24 h (Figure 6A, bottom panels). In addition, immunohistochemical results showed that the positive rate of ferroptosis-inhibiting molecule GPX4 decreased with the extension of reperfusion time in the ischemic areas of heart, and ferroptosis was significantly observed at 24 h of reperfusion (Figures 6B,D). Moreover, this was further confirmed by immunofluorescence (Figures 6C,E). As a result, ferroptosis is a key part in prolonged cardiac damage caused by I/R injury after 24 h reperfusion.

## 4 Discussion

PCD plays a crucial role in maintaining the normal structure of various cell types and heart function. It occurs in an orderly and controllable manner to preserve homeostasis, regulated by specific signaling pathways (Tang et al., 2019). Our research comprehensively described a spatiotemporal landscapes of various cell death forms in myocardial I/R injury, with an emphasis on accurately positioned single-cell interplay networks as well as their time point of appearance. Through integrated *in vitro* and *in vivo* experimental validations, our findings revealed that pyroptosis and apoptosis occur in the early stage of I/R injury. In contrast, necrosis and ferroptosis represent the predominant cell death modalities during prolonged reperfusion.

Thrombolytic and PCI therapies are crucial for restoring blood supply to the ischemic myocardium in patients with CAD. However, reperfusion can also induce I/R injury to the myocardium. In addition to destruction of the myocardium, it also can cause irreversible damage to the coronary microvasculature (Ibáñez et al., 2015). While multiple mechanisms have been investigated for their potential to alleviate cardiac I/R injury, effective drugs for the cardiac I/R injury have not been available. During the past 2 decades, several regulated modalities of cardiomyocyte death, including necroptosis, ferroptosis, pyroptosis, cuproptosis, apoptosis, and autophagy, have been identified in the context of I/R injury (Mishra et al., 2019; Davidson et al., 2020). Cardiomyocyte apoptosis emerged through the inherent pathway or extrinsic pathway, in response to DNA damage, increased the levels of ROS and cytosolic Ca^2+^, and activated sarcolemmal death receptors (Hengartner, 2000; Bernardi and Di Lisa, 2015). In the current study, reperfusion time was adopted as a variable to directly compare the apoptosis induced in mice after 6 h, 12 h, and 24 h of reperfusion. TUNEL positive cells began to appear after 6 h reperfusion, increased significantly after 12 h reperfusion, and a large number of apoptotic cells could still be observed after 24 h reperfusion. Cardiomyocyte apoptosis occurred in the early stage of reperfusion, and with the extension of reperfusion time, cardiomyocyte will continue to lose. Pyroptosis and necroptosis are characterized by impaired plasma membrane integrity, which elicits a pro-inflammatory response through the release of proinflammatory mediators such as interleukins and damage-associated molecular patterns (DAMPs) (Kawaguchi et al., 2011; Audia et al., 2018). We found that necroptosis, pyroptosis and necrosis mainly occurred in the middle and late stages of reperfusion. Studies have shown that the activation of autophagy does not lead to cell death during myocardial I/R injury but may play a protective role. Sala- Mercado, J. A. et al. found that in a pig model of I/R injury, activation of autophagy by chloramphenicol treatment reduced infarct size (Sala-Mercado et al., 2010). We investigated the occurrence of autophagy and discovered that it exhibited dynamic progression. Currently, the mechanisms and extent to which different forms of cell death interact in myocardial I/R injury remain unclear. However, specific target for each form of cell death has been shown to have an effect on cardiac infarct size. Koshinuma, S et al. found that inhibition of both necroptosis and apoptosis reduced infarct size obviously contrasted with suppression of either category of cell death just among isolated guinea pig hearts (Koshinuma et al., 2014). We focused on the time window in which ferroptosis took place and recognized the important part of ferroptosis in this present study. Ferroptosis is the predominant form of cell death in the prolonged reperfusion phase of myocardial I/R injury. During the reperfusion period, the burst of ROS leads to an imbalance in intracellular GSH/GPX4 antioxidant systems. As a result, this further promotes lipid peroxidation, more iron ions within the cell are involved in the Fenton reaction, and ferroptosis consequently continues to progress, worsening the myocardial injury. ACSL4, a key enzyme that regulates lipid composition, is activated to facilitate ferroptosis and myocardial I/R injury. Qiu et al. reported that inhibition of ACSL4 can effectively reduce myocardial I/R injury (Qiu et al., 2023). GPX4 protects myocardial cells against ROS damage-induced ferroptosis, inhibition of GPX4 leads to increasing mitochondrial ROS production and subsequent apoptosis in myocardial I/R injury (Xu et al., 2023). Based on coronary ligation models or isolated heart perfusion experiments, previous research has demonstrated that iron chelators can alleviate cardiac injury by inhibiting ferroptosis (Li et al., 2019; Fang et al., 2019; Fang et al., 2020). Furthermore, several key molecules involved in ferroptosis have been recognized as potential therapeutic targets for myocardial I/R injury. Accumulating evidence indicates that ferroptosis may participate in the prolonged phases of I/R injury. During the long-term reperfusion phase, both the combined application of ferroptosis inhibitors and targeted therapies against key molecules in the ferroptosis pathway hold the potential to maximize the efficacy of therapeutic strategies. This approach could effectively mitigate reperfusion injury and ultimately improve patient prognosis.

Reperfusion therapy has emerged as a cornerstone strategy for managing myocardial infarction, exerting substantial impacts on various cell types within the heart (Rentrop and Feit, 2015). Numerous studies have validated the occurrence of myocardial cell necrosis, endothelial cell dysfunction, macrophage polarization, and fibroblast proliferation (Molenaar et al., 2021). Single-cell sequence as an excellent way, construes the heterogeneity and intricacy of cell categories and subtypes involved in injury and the interrelationships. We conducted an analysis of single-cell sequencing data available in public databases in an attempt to clarify single-cell characterization of cell death forms in Myocardial I/R injury. We found that there was a substantial loss of cardiomyocytes after 24 h of reperfusion, with scores for all seven death modalities altered following reperfusion. Ferroptosis was particularly concentrated in cardiomyocytes, fibroblasts and macrophages. These results suggest that ferroptosis may play an important role. However, there are several limitations in our study. First, currently available public single-cell RNA sequencing datasets for mouse myocardial I/R models remain extremely limited, we only analyzed single cell sequencing data at 24 h of reperfusion and could not comprehensively reflect the changes in different modes of cell death at different time points, highlighting the need for additional single-cell sequencing studies to comprehensively investigate this aspect. Second, while our data revealed the observed changes in the mode of cardiomyocyte death at different times of hypoxia, we did not examine other cell types in the heart. These aspects require further investigation in future research. In addition, Cai et al. (Cai et al., 2023) reported a time-dependent decrease in the ferroptosis-inhibiting molecule GPX4 in mouse cardiac tissue following reperfusion, which contrasts with our observations in H9c2 cells. This discrepancy may stem from the relative insensitivity of H9c2 cells to ferroptosis detection, rendering them a suboptimal model for studying ferroptosis under H/R conditions. Future studies should employ isolated primary cardiomyocytes from murine heart tissue or neonatal rat cardiomyocytes, or alternatively, human-derived cardiomyocytes, to further validate these findings.

Although many studies have investigated the mechanism of different modes of cell death in myocardial I/R injury, such as apoptosis, necrosis, autophagy, ferroptosis, and pyroptosis (Del Re et al., 2019; Li et al., 2020; Heusch, 2020). The time window for the death of cardiomyocytes, fibroblasts, and macrophages during I/R injury remains unclear. Additionally, which pattern of cell death plays a more crucial role during different phases of myocardial I/R injury has not been thoroughly investigated. Cai et al. found that ferroptosis was the major category of cell death of cardiomyocytes following 1 day of reperfusion among mice, apoptosis and necroptosis caused cardiomyocyte death during the early phase of myocardial I/R injury by using inhibitors of different modes of cell death. However, it is important to note that experiments relying solely on inhibitors cannot accurately identify the critical cell death modality, as a single inhibitor may simultaneously affect signaling pathways of multiple cell death modes. In the current study, we clarified the relationship between cell death mode and reperfusion time. These findings may define the time window for therapy targeting different ways of cell death, thereby providing research directions and novel targets for exploring I/R injury treatments.

In summary, this analysis demonstrates that apoptosis takes place during the early stage of reperfusion. Besides, ferroptosis, necrosis and pyroptosis played a crucial role in the prolonged I/R injury period.

## Data Availability

The original contributions presented in the study are included in the article/Supplementary Material, further inquiries can be directed to the corresponding author.
