# Supplementary material for: Temporal regulation of genetic programs governing multiple cell death during myocardial ischemia-reperfusion injury
Source: Front Genet. 2025 Sep 5;16:1632867. doi: 10.3389/fgene.2025.1632867 (PMC12446000; doi:10.3389/fgene.2025.1632867)
Supplement: Supplementary file 1 [file DataSheet1.zip › Supplementary Material/Table S1-2.docx]

**Table S1**

**Antibody information**

| **P62** | **HUABIO(#EM0704)** |
| --- | --- |
| **GSDMD** | **HUABIO(#HA721144)** |
| **GPX4** | **HUABIO(#ET1706-45)** |
| **LC3** | **CST(#4108s)** |
| **GAPDH** | **HUABIO(#ET1601-4)** |
| **BCL2** | **HUABIO(#ET1702-53)** |

**Table S3**

**Primer sequences**

| **RAT-*Ripk3*-R2** | **GGATTGCTGTTTCTTCCATC** |
| --- | --- |
| **RAT-*Ripk3*-F2** | **TTCCACATACTTTACCCTCCA** |
| **RAT-*Hsp70*-R2** | **GGCTGTTGCTCTTCAGTATGT** |
| **RAT-*Hsp70*-F2** | **GCATTTTCAGTGTGTCCAGT** |
| **RAT-*Acsl4*-R2** | **TGTCCATTTTCATCCACAGA** |
| **RAT-*Acsl4*-F2** | **GATAAGCCAAACCCCAGAG** |
| **RAT-*Gpx4*-R1** | **CGCAGCCGTTCTTATCAAT** |
| **RAT-*Gpx4*-F1** | **CTGTGTAAATGGGGACGATG** |
| **RAT-*Atg5*-R2** | **ACAGGACGGAACAGCTTCT** |
| **RAT-*Atg5*-F2** | **ATGATTTGACCAGTTTTGGAC** |
| **RAT-*Bcl2*-R2** | **GCTGGAAGGAGAAGATGCC** |
| **RAT-*Bcl2*-F2** | **ACGAGTGGGATACTGGAGATG** |
| **RAT-*Bax*-R2** | **CCAGTTGAAGTTGCCGTCT** |
| **RAT-*Bax*-F2** | **GAGGATGATTGCTGATGTGG** |

| **MUS-*Ripk3*-R** | **CTGTGCTTGGTCATACTTGG** |
| --- | --- |
| **MUS-*Ripk3*-F** | **GGCTCTCGTCTTCAACAACT** |
| **MUS-*Hsp70*-R** | **CCGCTGAGAGTCGTTGAAG** |
| **MUS-*Hsp70*-F** | **ATGGTGCTGACGAAGATGAA** |
| **MUS-*Acsl4*-R** | **GTTGGTCTACTTGGAGGAACG** |
| **MUS-*Acsl4*-F** | **CCTGAGGGGCTTGAAATTCAC** |
| **MUS-*P62*-R** | **GCTGCGACAGGATAAAGTCCA** |
| **MUS-*P62*-F** | **TCCAGCGATGACCTATGCAC** |
| **MUS-*Atg5*-R** | **CACTTTGTCAGTTACCAACGTCA** |
| **MUS-*Atg5*-F** | **AGTCAAGTGATCAACGAAATGC** |
| **MUS-*Bcl2*-R** | **CCCCACCGAACTCAAAGAAGG** |
| **MUS-*Bcl2*-F** | **GCTACCGTCGTGACTTCGC** |
| **MUS-*Bax*-R** | **TGTCCAGCCCATGATGGTTC** |
| **MUS-*Bax*-F** | **AGGAGAGACCGCTTAGCAGA** |
