# Supplementary figures and images for: Temporal regulation of genetic programs governing multiple cell death during myocardial ischemia-reperfusion injury
Source: Front Genet. 2025 Sep 5;16:1632867. doi: 10.3389/fgene.2025.1632867 (PMC12446000; doi:10.3389/fgene.2025.1632867)

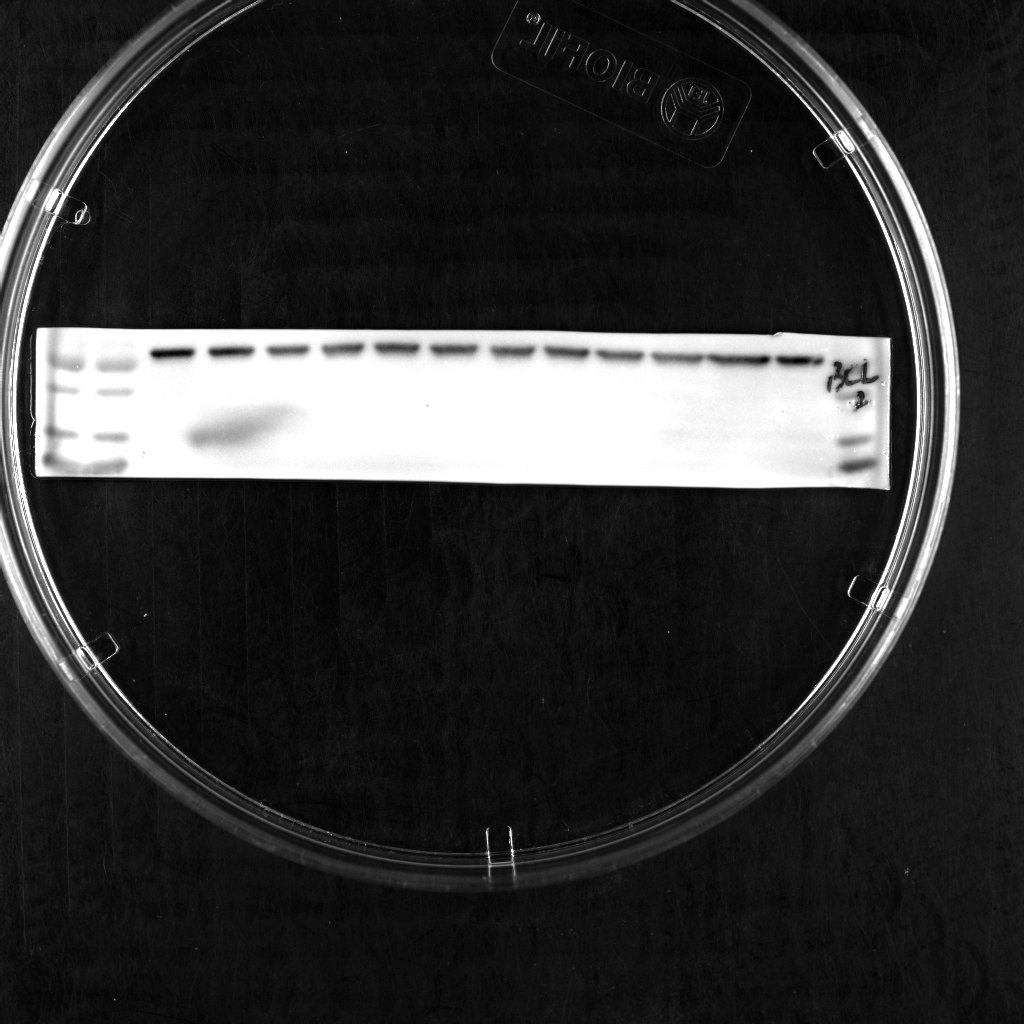

Supplement: Supplementary file 1 [file DataSheet1.zip › Supplementary Material/GelsBlots/BCL2.Tif]

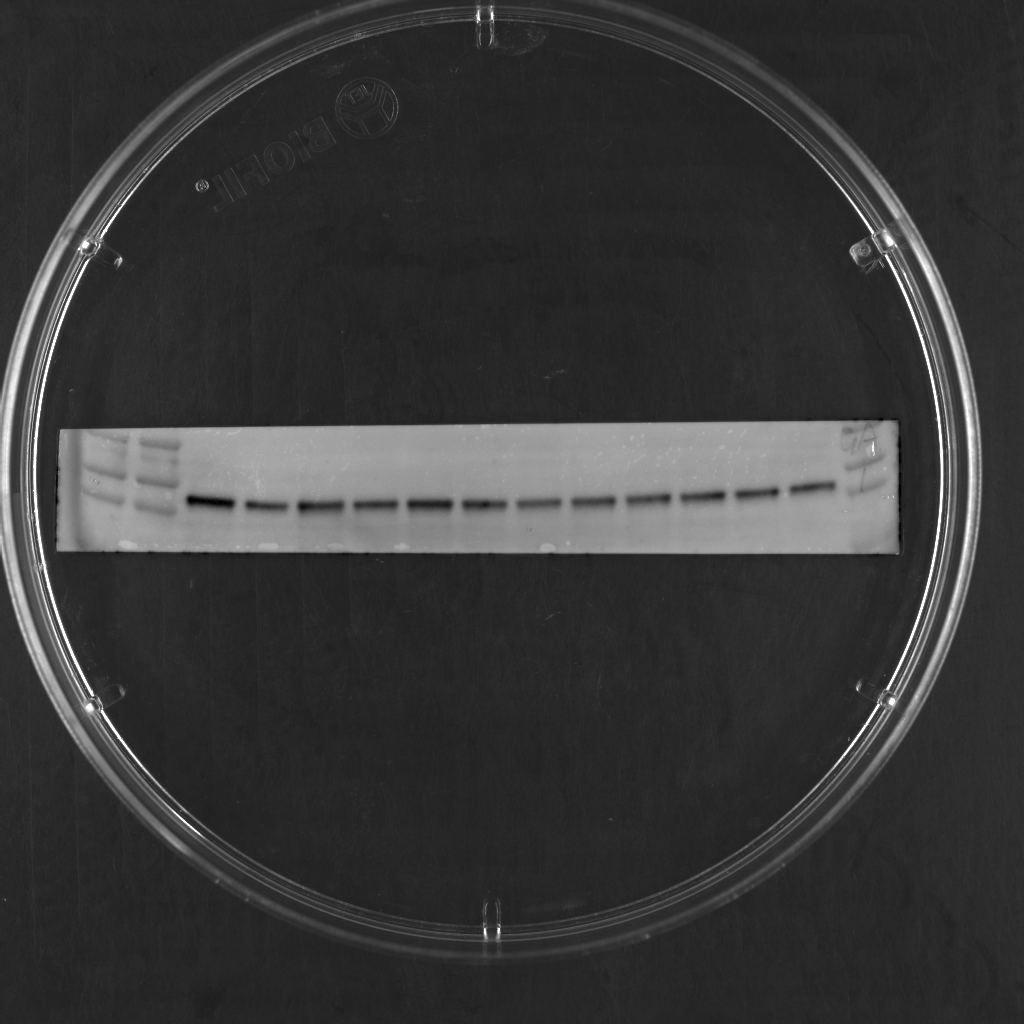

Supplement: Supplementary file 1 [file DataSheet1.zip › Supplementary Material/GelsBlots/GAPDH1.Tif]

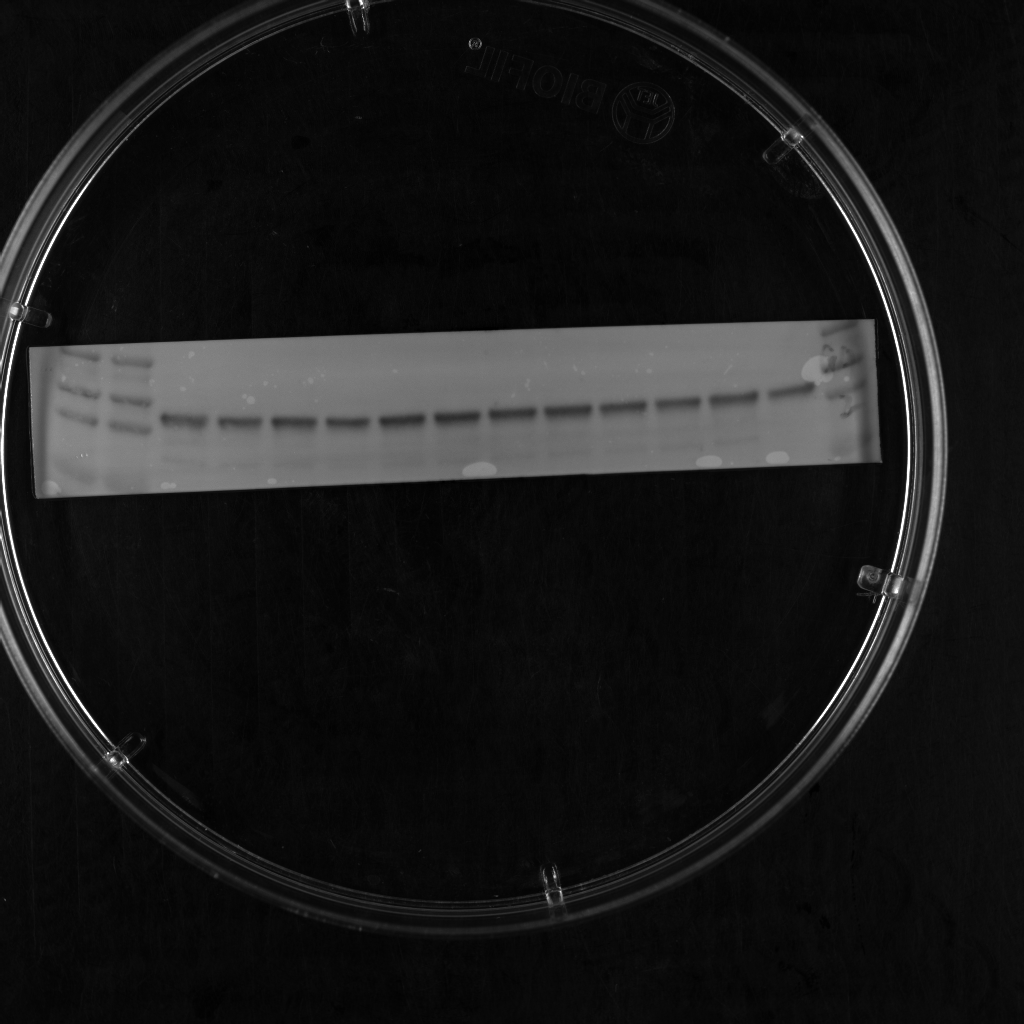

Supplement: Supplementary file 1 [file DataSheet1.zip › Supplementary Material/GelsBlots/GAPDH2.Tif]

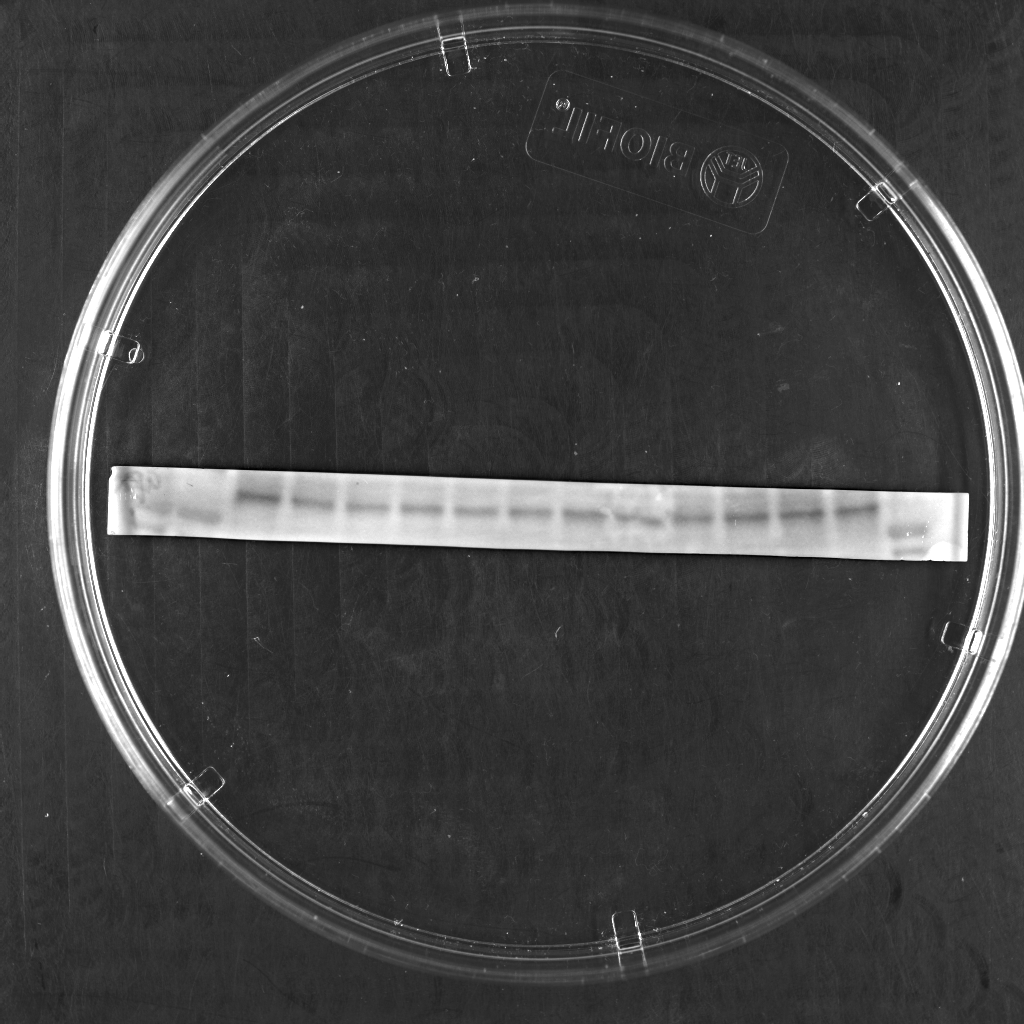

Supplement: Supplementary file 1 [file DataSheet1.zip › Supplementary Material/GelsBlots/GPX4.Tif]

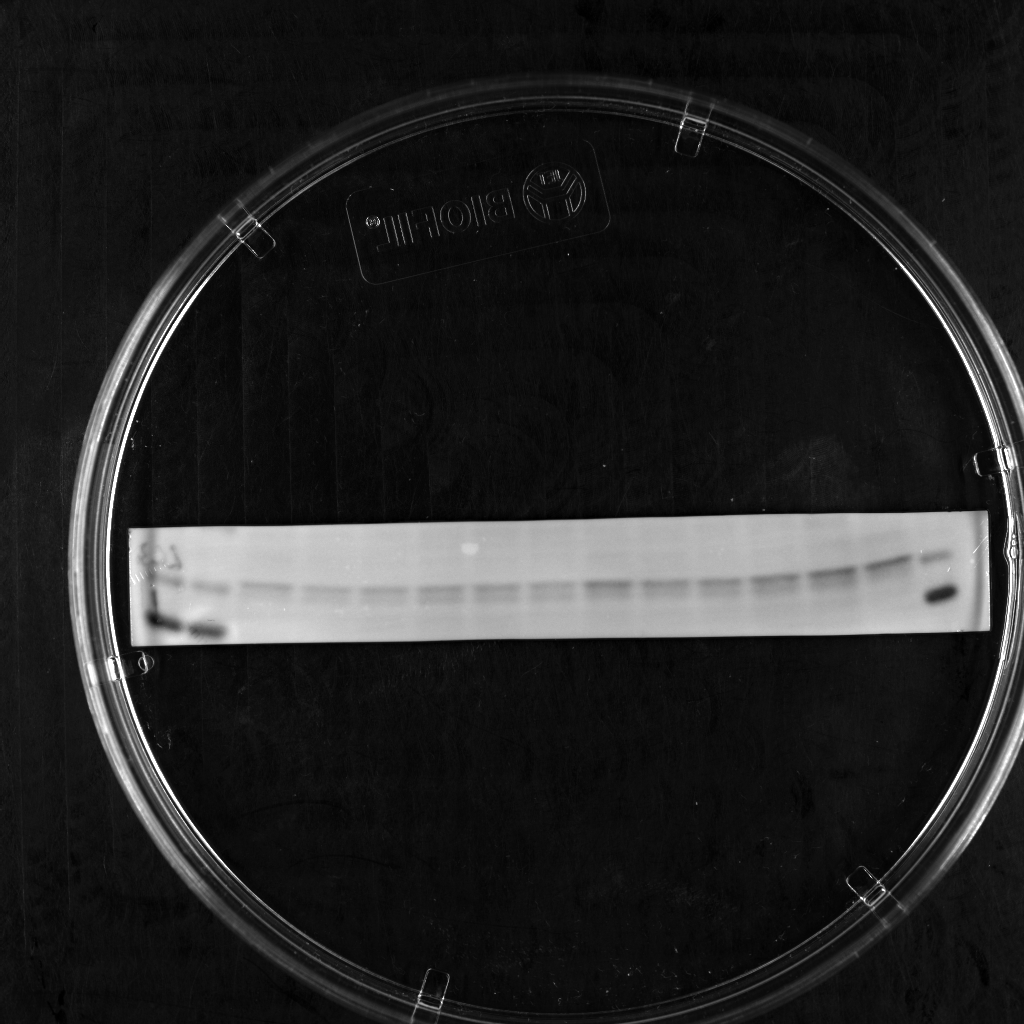

Supplement: Supplementary file 1 [file DataSheet1.zip › Supplementary Material/GelsBlots/LC3.Tif]
